# ERK pathway agonism for cancer therapy: evidence, insights, and a target discovery framework

**DOI:** 10.1038/s41698-024-00554-5

**Published:** 2024-03-14

**Authors:** Oleg Timofeev, Philippe Giron, Steffen Lawo, Martin Pichler, Maxim Noeparast

**Affiliations:** 1 Institute of Molecular Oncology, Member of the German Center for Lung Research (DZL), Philipps University, 35043 Marburg, Germany; 2 Vrije Universiteit Brussel (VUB), Universitair Ziekenhuis Brussel (UZ Brussel), Clinical Sciences, Research group Genetics, Reproduction and Development, Centre for Medical Genetics, Laarbeeklaan 101, 1090 Brussels, Belgium; 3 CRISPR Screening Core Facility, Max Planck Institute for Biology of Ageing, 50931 Cologne, Germany; 4 Translational Oncology, II. Med Clinics Hematology and Oncology, 86156 Augsburg, Germany

**Keywords:** Targeted therapies, Oncogenes

## Abstract

At least 40% of human cancers are associated with aberrant ERK pathway activity (ERKp). Inhibitors targeting various effectors within the ERKp have been developed and explored for over two decades. Conversely, a substantial body of evidence suggests that both normal human cells and, notably to a greater extent, cancer cells exhibit susceptibility to hyperactivation of ERKp. However, this vulnerability of cancer cells remains relatively unexplored. In this review, we reexamine the evidence on the selective lethality of highly elevated ERKp activity in human cancer cells of varying backgrounds. We synthesize the insights proposed for harnessing this vulnerability of ERK-associated cancers for therapeutical approaches and contextualize these insights within established pharmacological cancer-targeting models. Moreover, we compile the intriguing preclinical findings of ERK pathway agonism in diverse cancer models. Lastly, we present a conceptual framework for target discovery regarding ERKp agonism, emphasizing the utilization of mutual exclusivity among oncogenes to develop novel targeted therapies for precision oncology.

## Background

### Cancer targeting: from chemotherapy to precision oncology

**Fig. 1:**
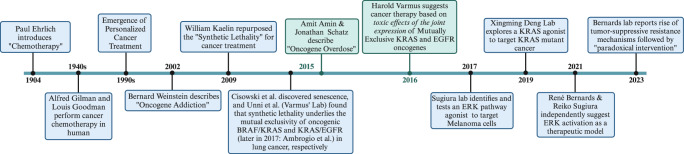
Timeline of cancer-targeting discoveries and concepts relevant to this writing. The focus of the timeline is mainly on therapeutics targeting the cancer cells. Figure generated in Biorender.

In 1904, after a series of studies on different model organisms, Paul Erlich, a highly meritorious German medical scientist, coined *chemotherapy* to address his approach when chemicals were used to target infectious diseases^1,2^ (see Fig. 1). Ehrlich humbly stated: *Indeed, from the very origin of the art of healing, chemotherapy has existed since almost all the medicaments we employ are chemicals*^1^. However, Ehrlich’s concept of chemotherapy unprecedently linked the chemical structure and chemoreceptors on the target cells to make sense of the pharmacological activity^3,4^. He addressed chemicals that selectively exert their detrimental effect on the target cells (e.g., parasites) and not on the treated organism cells as *Zauberkugeln* or magic bullets^1,2^. Erlich had tested compounds, such as alkylating agents, to target cancer, which, in his hands, showed limited effects, far from being considered Zauberkugeln. Still, Ehrlich remained optimistic that effective chemotherapeutics could be explored in the future^1^. He explained this lack of effect by the high similarity between cancer and non-cancer cells^1^.

During World War II, in a mouse xenograft lymphoma model, two American pharmacologists, Alfred Gilman, and Louis Goodman, showed that nitrogen mustard, a warfare gas, had (chemo)therapeutic effects^5,6^. Their work immediately justified the treatment of a non–Hodgkin’s lymphoma patient and other cancers with nitrogen mustard^5,7^. Despite further knowledge about its limited efficacy and proneness to resistance, nitrogen mustard’s ephemerous success laid the foundation for establishing cancer chemotherapy as a valid field. Since then, several cancer chemotherapeutics such as Alkylating, Antimicrotubular agents, and Antimetabolites have been developed^8^. Cancer chemotherapy is a term whose definition was progressively elucidated long after it was coined, considering the expanding knowledge about chemotherapeutics’ mechanism of action and limitations. Compared to more selective therapeutics, Chemotherapeutics are considered one-size-fits-all treatments targeting different cancer types carrying distinct driver oncogenes^8^. Despite their narrow therapeutic window and the possibility of targeting non-cancerous dividing cells, chemotherapeutics have saved lives in some cancer populations, such as pediatric hematologic cancers, or among adult malignancies, such as testicular cancer^9^. Until today, chemotherapy, surgery, and radiotherapy are on the list of available neoadjuvant, adjuvant, or combined cancer treatments^8,10^.

Followed by the burst of knowledge about cancer-related gene mutations in the 1990s, targeting oncogenes laid the foundation for targeted therapy in cancer^11^. Indeed, since the *New Era of Personalized Medicine* in 1999^11^, cancer treatment has been revolutionized. We have seen the mainstream shift from “one‐size‐fits‐all” approaches, such as classical chemotherapy, to individualized treatments according to patients’ tumor genetic profiles^12,13^. A milestone in personalized cancer therapy was the success of Imatinib, a small molecule inhibitor of the BCR‐ABL protein tyrosine kinase. This inhibitor was initially conceptualized and profiled by Nicholas Lydon and colleagues^14^. Imatinib demonstrated remarkable clinical advantages during phase II trials, particularly among patients with chronic myeloid leukemia (CML)^15,16^.

By 2002, to explain mechanisms behind the sensitivity of some cancers to mutation-specific targeting, Bernard Weinstein coined the term *oncogene addiction* to describe a phenomenon that, despite extensive genetic alterations, cancer cells can depend on a single oncogene activity^17–23^. One can analogize eliminating that very oncogene activity in the addicted cell to surpassing the cell’s system biology threshold, ultimately leading to a lethal trade-off favoring proapoptotic vs. the prosurvival signals. Various therapeutics have been developed against other oncogenes to which different cancers are addicted. EGFR- or ALK-altered lung cancers and BRAF mutant melanoma are some success stories of personalized cancer treatments^24–26^. All these therapeutics work by inhibiting a driver oncogene or its downstream effector, based on the rationale that withdrawal from oncogene addiction leads to deleterious *oncogenic shock*^27^. Indeed, target inhibition is a shared feature of all these treatments. Although the targets are often kinase, non-kinase protooncogenes, such as KRAS^G12C^, are also actionable^28^. Sooner or later, however, all these treatments are doomed to the rise of resistance mechanisms, leading to the relapse of the treated cancer^25,29–33^.

A theoretical alternative of oncogene inhibition as a therapeutic concept is the re-introduction or restoration of a lost tumor suppressor (TS) activity, as cancer cells might be sensitive to that lost function^34,35^. However, this strategy has been proven challenging as often, if not always (e.g., some p53 variants), TS genes are lost due to various genetic and epigenetic alterations and, therefore, non-targetable in cancer cells. The p53 targeted therapies, conceptualized according to the p53 status of the cancer cell, are being explored^36^. These approaches can involve different strategies, from stabilizing the wild-type p53 and unleashing its wide range of TS functions in cells with existing wild-type copies to restoring the wild-type conformation among responsive mutant p53 variants^36^. In the bargain, numerous efforts have been made to re-express tumor suppressors by viral and non-viral gene therapy in tumor cells^37^. Viral tumor suppressor gene transfer has even been combined with the TS-loss-targeted-oncolytic capacity of therapeutic viruses or synergistic effects of TS re-expression with immunotherapeutics to double-punch the cancer cells^37–40^. Despite all these cumbersome efforts, none has yet borne fruit in the clinic among large patient populations. Moreover, in the late stages of cancer, due to the highly divergent genetic build-up of late-stage tumor cells, it is to be further elucidated whether cells have already circumvented their native sensitivity and response to the lost tumor suppressors.

By 1945, Theodosius Dobzhansky, a Russian-American geneticist, had coined the term *synthetic lethality*, describing a scenario when two distinctive chromosomes in *Drosophila pseudoobscura* were tolerated if existing alone but became lethal when they co-existed synthetically, as being imposed in the lab, in cross-over flies^41^. In 2009, Nobel Prize laureate William Kaelin Jr. put synthetic lethality forward as a conceptual framework for exploring novel anticancer treatments^42^. The cancer research field further translates this as the genetic build-up in certain cancers conferring sensitivity to specific chemical or genetic perturbations, opening novel avenues for indirectly targeting previously non-druggable targets^43,44^. Today, synthetic lethality is described as the cellular lethality of at least two co-occurring contextual (genetic) or chemical perturbations targeting at least two separate genes. In contrast, these perturbations are tolerated at variance^45^. As a result, within the scope of personalized medicine, treatments have been developed by targeting specific non-oncogenes based on the concept of synthetic lethality^43,44^. An example of such an approach in the clinic is the adjuvant as well as palliative PARP inhibitor treatment of BRCA1/2-mutant HER2-negative breast cancer^46^. An exciting feature of synthetic lethality is that it can be applied to both an oncogene and a tumor suppressor as long as they confer sensitivity to a synthetically lethal target^44^. An interesting reconceptualization of synthetic lethality as a cancer-targeting approach, namely the *one-two punch*^47^ model, is being pioneered and explored by René Bernards and colleagues. In this model, the first-line treatment is designed to induce senescence-associated vulnerabilities in cancer cells that are to be targeted with the second-line *senolytic* compounds^47^.

Notably, in recent years, targeting cancer-related immune cells or their interaction with cancer cells has opened a new front in the battle against cancer^48–50^. For instance, in BRAF mutant melanoma, in which targeted inhibitors are known to be at their highest performance, immune checkpoint inhibitors showed a 20% benefit over targeted compounds in terms of overall survival among therapy-naïve and metastatic patients^51^. As such, oncogene-targeted therapy might seem on the verge of losing momentum in personalized medicine. However, despite the striking responses to different cancer immunotherapeutics in a group of patients, many patients remain non-responsive or fast-resistant to such therapies^48–50,52^.

While it is essential to work towards enhancing existing therapeutic strategies, it is also highly justified to put forward and explore innovative and unconsumed concepts.

By 2015, two significant studies showed that mutual exclusivity among BRAF/KRAS and EGFR/KRAS oncogenes can result from synthetic lethality or senescence^43,53^.

The British fairy tale “*Three Bears*” (Eleanor Mure, 1831) revolves around a *greedy* individual who, in the absence of the three bachelor bears, each of varying sizes, enters their cottage^54^. She samples their trio of distinct milk portions, chairs, and beds. Repeatedly, amidst these triads, she discovers contentment solely when the element is precisely balanced – not too much, not too little, but just right^54^. In later biological and medical contexts, the widely spread but modified version of the original story “*Goldilocks and the Three Bears*,” in which a young girl replaces the elderly woman, has inspired the *Goldilocks principle*. This principle signifies that, for a biological system to function optimally, its components should possess the correct levels of abundance and activity and be temporally right – avoiding both excess and deficiency^55^.

Amit Dipak Amin and Jonathan Schatz, inspired by the Goldilocks principle in Biology, introduced the term and the concept of “*Oncogene Overdose*.” This concept compares cancer cells to drug addicts, as both can die due to an overwhelming excess of what they are addicted to^55^.

In 2016, Nobel laureate Harold Varmus introduced the concept of harnessing mechanisms elicited by the co-induction of mutually exclusive genes as a therapeutic model^56^. Furthermore, Xu et al. from Deng’s laboratory have explored a striking KRAS agonist for targeting KRAS mutant cancers^57^. More recently, Sugiura et al. presented the therapeutic concept of ERKp activators^58–60^. Concurrently, René Bernards and colleagues described *Paradoxical Intervention*, signifying the simultaneous activation of mitogenic signals and the employment of stress-inducing compounds in cells with pre-existing ERK pathway-activating alterations^61,62^. Remarkably, they have demonstrated that such treatment enforces unprecedented tumor-suppressive resistance mechanisms^62^.

Dostoevsky once remarked, *‘We all come out from Gogol’s ‘Overcoat*,” paying homage to Nikolai Gogol as the iconic predecessor of Russian literature preceding his own generation. In doing so, he employed wordplay by referring to one of Gogol’s renowned short stories, namely, ‘*The Overcoat*.’“ Based on chronological order and logical reasoning, all the above concepts revolve around over-activating the already activated pathway as a therapeutic approach, akin to stepping out from oncogene overdose overcoat.

In this writing, we review the evidence and insights that reinforced ERK pathway effectors activity in cells with aberrantly increased ERK activity is damaging to these cells. The pathway is also known as mitogen-activated protein kinase (MAPK) or Ras-Raf-MEK-ERK pathway. In this writing, for simplicity, we will mention the pathway by its major effector ERK without distinguishing between the two human ERK genes, ERK1 and ERK2. Furthermore, we will present a conceptual framework for therapeutic targeting of such vulnerabilities.

### The double-life of ERKp in human cancer and the curious case of the ERK proteins

The ERK pathway is one of the extensively studied signal transduction cascades. Essential effectors of this signaling cascade in humans fall into three types of eukaryotic protein kinases: (1) HER family Receptor Tyrosine Kinases (RTKs, EGFR, HER2-4), RAF family serine/threonine kinases (A/B/CRAF), and (3) dual specificity protein kinase families MEK (MEK1/2) and ERK (ERK1/2)^63–65^. In humans, the RAS family, consisting of three essential members, H/N/KRAS, serve as the principal non-kinase effectors within the ERK pathway^66^.

Feedback loops regulate ERK signaling during development and in normal physiologic conditions for cell proliferation, survival, and homeostasis^63–66^.

Due to genetic alterations of the ERK pathway effectors and/or its regulatory components, ERK signaling may become pathologically deregulated. Aberrant down-regulation of the ERK pathway can be associated with some neurodegenerative or autoimmune disorders, while its abnormal upregulation is associated with human cancers, rasopathies, and Erdheim-Chester disease^66–69^.

Under normal cellular conditions, upon ligands binding, HER receptors can undergo conformational changes, transitioning to an active state and forming homo- or hetero-dimers^70^. The induced conformational alterations in the tyrosine kinase domain of dimerization partners facilitate ATP binding in their ATP binding cleft and, as suggested, in the case of EGFR, might lead to the relief of cis-autoinhibition and trans-autophosphorylation of the tyrosine kinase domains^70,71^.

Under physiological circumstances, the mature RAS protein, when residing in the inner surface of the plasma membrane, undergoes conformational changes mediated by the upstream HER signaling or regulatory feedback signals^66,72^. RAS alternates between a guanosine triphosphate (GTP)-bound state and a guanosine diphosphate (GDP)-bound state^28,66,73^. The oscillation of RAS into its active conformation involves GTP loading^28,66,73^. This process can be initiated by recruiting GRB2 proteins to the plasma membrane, where the GRB2 is activated via its SH2 domain by an active RTK^66^. Subsequently, SOS is recruited, forming a complex involving at least GRB2, SOS, and RAS^66^. This complex facilitates the displacement of GDP and promotes the loading of GTP onto the RAS molecule^66^. RAS can hydrolyze its GTP as a GTPase protein, converting it to GDP and becoming inactive^28,66,73^. This process is facilitated by GTPase-activating proteins (GAPs)^28,66,73^. Feedback loops play a regulatory role in the GTPase activity of RAS^66^. The GTP-bound RAS triggers the activation of downstream RAF by engaging in a physical (allosteric) interaction with RAF or mediating the release of its autoinhibition^74^. RAFs, in turn, phosphorylate the MEK1/2 through phosphotransferase activity, and MEKs, in turn, phosphorylate the ERKs, which, due to the Erks’ vast array of network, major cellular events such as proliferation and survival can take place^63–66^. At least 659 of ERKs’ direct substrates are discovered^75^.

Approximately 40% of human cancers are linked to the abberant upregulation of ERKp^76^. Notably, cancers that harbor genetic alterations in an ERKp effector can be associated with worse clinical outcomes than cancers where such alterations are absent, emphasizing the significance of these alterations on cancer prognosis^77^. For over two decades, the effective inhibition of the ERKp has remained a key focus in cancer research and targeted therapy efforts^12,24,26,28,77^.

**Fig. 2:**
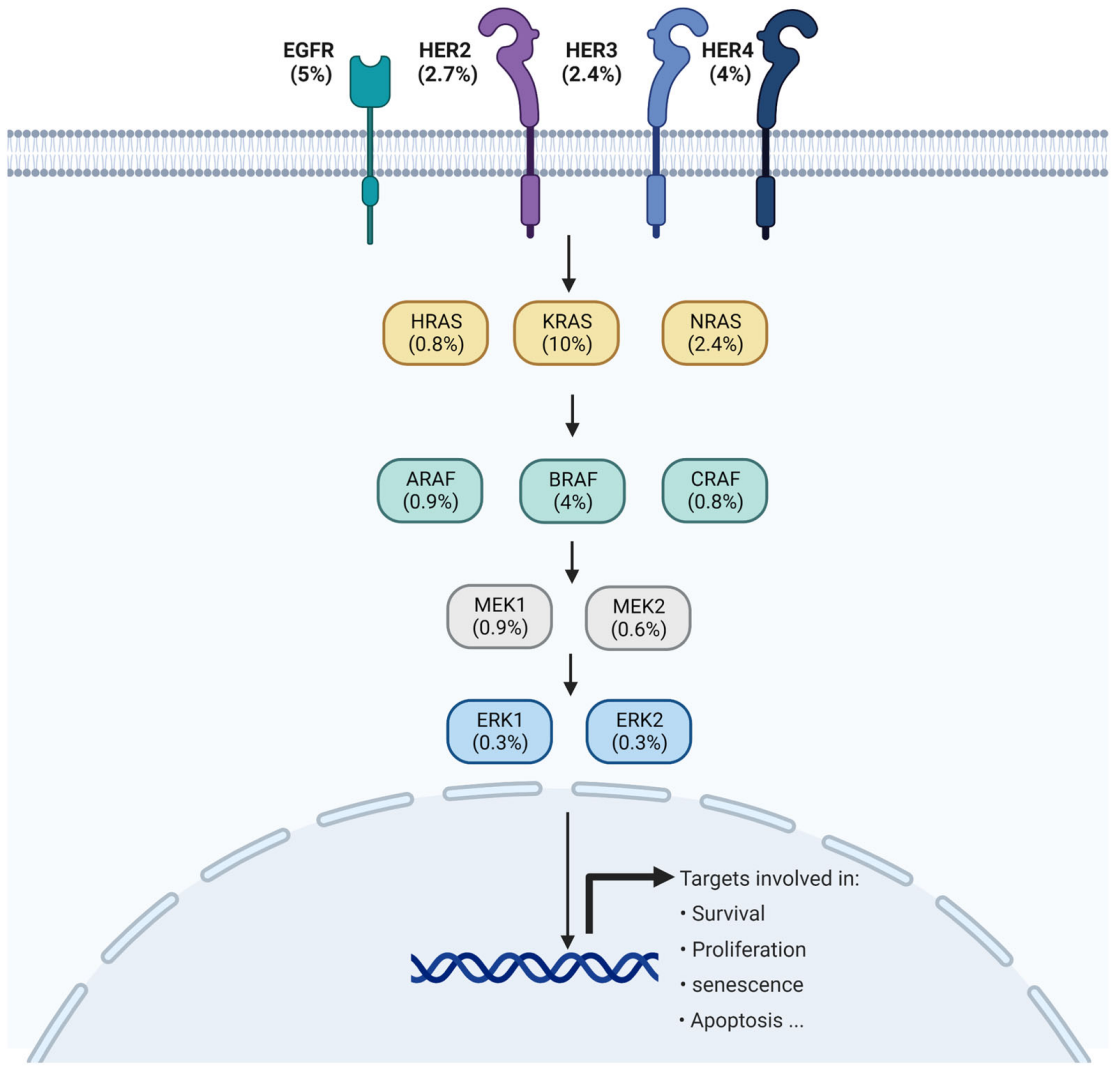
ERK1/2 mutations are relatively rare. In July 2023, the query of 69223 samples belonging to 65853 patients in 213 curated and non-redundant studies in cBiportal for only mutations among conventional effectors of the ERKp yielded the approximate frequencies as displayed. Note that this oversimplified schematic is not meant to communicate complex signaling and structures of the effectors. Unlike typical oncogenes, ERK1/2 mutations are distributed along the ERK protein’s conserved protein kinase and non-conserved regions, with no hotspot mutational site. Figure generated in Biorender.

However, in an ironic twist, a wealth of evidence underscores the reality that human cells, including cancer cells, cannot tolerate excessive levels of ERKp activity. While upstream effectors of the ERKp, such as RTKs, RAS, and RAFs, are subject to activating and oncogenic mutations in humans, the mutations in downstream effectors, such as MEK and even to a greater extent ERK, are infrequent^26,73,77,78^. Before the ERK small molecule inhibitors’ development and clinical employment, ERK mutations were hardly reported in human cancer^79^. These mutations can be associated with secondary resistance mechanisms that lead to loss-of the protein affinity to ERK inhibitors in cells treated with these compounds^79–81^. As illustrated in Fig. 2, among the conventional ERK signaling pathway effectors, the ERK mutations still rank at the bottom of the list regarding the frequency of mutations in human cancer.

The ERKs need two critical phosphorylation events before full conformational activation, none triggered by either cis- or trans-autophosphorylation mechanisms^82^. As opposed to RTKs, RAFs, or even MEKs, ERKs protein conformations have evolved in such a way to be less poised for autoactivation and instead rely on direct phosphotransferase activity of MEKs for full activation^82,83^.

Several activating variants that cause increased kinase-dependent or -independent activity of the ERK orthologues, such as *Sevenmaker* variants, are studied in Yeast and Drosophila models^83–85^. However, how such mutations can induce or contribute to human carcinogenesis remains enigmatic. Several investigations have concentrated on characterizing a few purportedly activating ERK variants by expressing them in mammalian cells^82,86,87^. These studies have looked into activating variants of ERK that render the ERK protein an autoactivation capacity^82,86,87^. In particular, the activating variant ERK1^R84H^, reported twice in human cancer, was found to transform NIH3T3 cells^87^. While introducing these presumably activating ERK variants into non-cancerous mammalian cells was occasionally feasible, accomplishing the same in human cancer cell lines appeared to pose challenges. Markedly, Goetz et al. had shown in the past that overexpression of wild-type ERK1/2 and not the kinase-dead or low-kinase ERK1/2 variants in BRAF^V600E^ mutant melanoma cell line A-375 leads to growth inhibitory effects^88^. Interestingly, the ERK1/2 variants with increased kinase activity, which were found to confer resistance against RAF inhibitors (RAFi) and MEK inhibitors (MEKi) during a random mutation screen, even exerted more potent growth inhibition when overexpressed in A-375 cells. The most active ERK1/2 variants exhibited growth-suppressive effects in two other BRAF mutant melanoma cell lines beyond A-375 cells(SKMEL-19, and WM266.4)^88^. It was also shown that inducible overexpression of ERK2 in A-375 cells was selectively detrimental to these cells vs. cells with wild-type BRAF and caused anti-tumor effects in vitro and in vivo^89^. Such detrimental effects could only be rescued upon ERK2 or BRAF knock-down^89^. The ERK2 overexpression in these cells was associated with the induction of ER stress and DNA damage in addition to proapoptotic signals^89^. The fact that some ERK-associated cancers, such as BRAF mutant melanoma, are sensitive to ERK activation is well-established.

Interestingly, the negative effect of putative Gain-of-Function (GOF) mutations on the proliferation of these cells has been utilized as a model in an ERK saturation mutagenesis study^90^. Such a comprehensive approach by Brenan et al. has shed light on the relevance of ERK mutations in human cancer. It is worth noting that saturation mutagenesis of ERK was unsuccessful in identifying the direct GOF impact of activating variants, unless upon MEKi and BRAFi treatment. Indeed, expression of GOF sevenmaker ERK mutations ERK2^D321N^ and ERK2^E322K^ or other supposedly GOF variants in the A-375 cell line had a robust anti-proliferative effect in these cells^90^. Interestingly, the sevenmaker variant and those that phenocopy its effect could only be tolerated by the BRAF^V600E^ cells under MEK or RAF inhibition^90^. These variants are proposed to enhance ERK activity by disrupting ERK’s interaction with inhibitory DUSP phosphatases^90^.

The rarity of ERK mutations in human cancer raises an intriguing question, prompting consideration of at least two alternative scenarios to explain this phenomenon.

One possibility is that the ERK protein, by default, exhibits low basal activity and has a weak impact on its network. Consequently, even an activating mutation might not be sufficient to manifest an effective GOF phenotype leading to cell transformation. An illustrative example of such a scenario is the case of CRAF mutations in human cancer, which are rarer than mutations of another RAF isoform, BRAF^91,92^. Past explanations attribute this rarity to the low basal activity of CRAF compared to BRAF^93^. Conversely, the effectors, such as EGFR, which are more upstream and have a wider array of targets belonging to distinctive pathways, exhibit a higher frequency of mutations in human cancer. From a vertical signaling cascade standpoint, the three commonly mutated effectors of the ERKp in human cancer are arranged as EGFR, KRAS, and BRAF. EGFR and BRAF mutations exhibit a comparable frequency in human cancer (see Fig. 2). Notably, KRAS mutations occur at a frequency equivalent to the combined occurrences of EGFR and BRAF oncogenes (Fig. 2). The higher mutational frequency of KRAS over BRAF can be explained by its vertical rank along the signaling cascade, a rationale not applicable to the other two effectors^82^.

Another explanation could be that while ERK mutations capable of generating an effective GOF phenotype may exist synthetically, they are negatively selected in the real world due to being poorly tolerated by human cells. In contrast to, for instance, KRAS, which oscillates between active and inactive states under normal physiological conditions and may become constitutively active in human cancer^73^, human cells cannot tolerate ERKs in a constitutively active state. It is crucial to distinguish between two types of ERK mutations. The first type comprises mutations that occur in the real world. As the above evidence suggests, these mutations exhibit low transforming capacity. Then comes the synthetic ERK mutations, which do not occur in the real world. Indeed, as mentioned in the above paragraphs, such synthetic variants have been studied in the past, and it has been shown that they are not easily tolerated by human cells, particularly cancer cells^79,81,82,84–86,88–90^. More interestingly, in addition to some ERK mutants, even wild-type ERK overexpression is not tolerated in such human cellular models^88,90^. Indeed, one of the vital pieces of evidence is ERK saturation mutagenesis by Brenan et al., as they observed in human melanoma cell line model with BRAF^V600E^, expression of wild-type ERK and activating ERK variants could be tolerated only in the presence of ERK pathway inhibitors^90^. This evidence, at least, can rule out the possibility that ERK, by nature, cannot render gain-of-function. The scenario that ERK-activating mutations are not easily tolerated by human cells aligns with one aspect of ERK protein activity autoregulation, namely its reduced potential for autoactivation, in contrast to other ERK pathway effectors like EGFR^82,83^. The ERK protein is shown to tolerate synthetic mutations that can render it increased autophosphorylation and kinase activity to levels comparable to ERK5^82^.

The RTK/RAS/RAF pathway and its inhibitors exhibit a Janus-faced immunomodulatory effect in cancer (reviewed here^94^). In brief, inhibition of the ERKp can enhance the activity of associated T-helper cells and cytotoxic T-cells, thereby potentially enhancing the immune response against cancer cells^94^. Additionally, it can enhance dendritic cell activity, which plays a crucial role in antigen presentation and immune activation. Conversely, this inhibition may hamper tumor infiltration of immunosuppressive regulatory T-cells, monocytes, and macrophages, potentially limiting their immunosuppressive functions^94^. On the other hand, long-term ERKp inhibition might eventually have an immunosuppressive effect^95^. Of note, oncogenic RAS can contribute to immune evasion by stabilizing the PD-L1 mRNA and subsequently favoring the PD-L1 expression on tumor cells^96^.

### ERK pathway hyperactivation can be lethal to cells in various ways

**Fig. 3:**
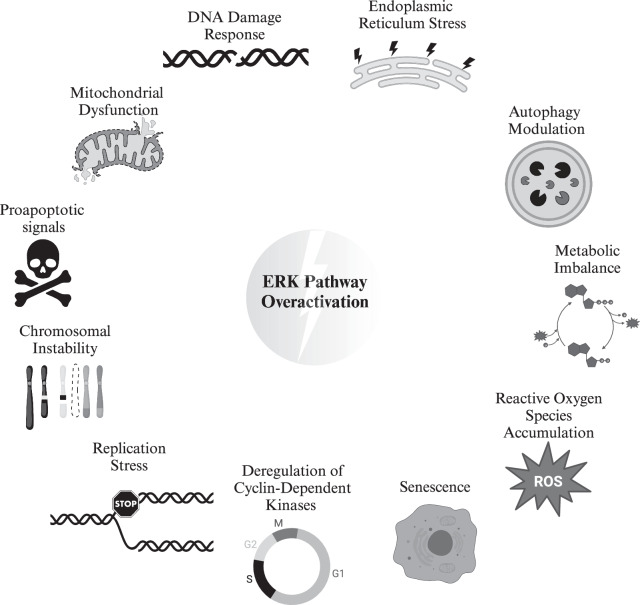
Intertwining mechanisms by which excessive ERK activity leads to cell damage. Figure generated in Biorender.

The ERKp activity can lead to cell toxicity and death (see Fig. 3). Numerous studies have unraveled such a role in different model organisms^60,97,98^. The ERK-induced cell apoptosis can involve extrinsic or intrinsic apoptotic pathways^60,97,99^. ERK activity affects cell death by influencing multiple cellular processes, including mitochondrial dysfunction^100–102^, DNA Damage Response^103^, Endoplasmic Reticulum stress^104^, Autophagy modulation^60,105^, Metabolic imbalance^106^, and accumulation of Reactive Oxygen Species(ROS)^97,103^. ERKp activity can trigger senescence in vitro and in vivo^97^. An interesting example of such a phenomenon is the existence of benign human naevi with BRAF^V600E^ mutation^107,108^. In cases where this mutation occurs in isolation and without the concurrent loss-of the p53 tumor suppressor, it has been proposed to result in irreversible cellular senescence rather than malignancy^108^. More recently, an alternative mechanism involving non-coding RNAs has come to light, linking BRAF^V600E^-carrying benign naevi to occurrences of mitotic failure and reversible proliferation arrest^107^.

ERKp activity is crucial in determining divergent cellular outcomes, including cell proliferation, growth arrest, or cell death. Notably, Hong et al. from Park’s lab have elucidated an exclusive threshold for ERK activity^109^. Previously, it was known that excessive activity of ERKs, like other effectors such as MEKs, CRAF, and BRAF, can cause growth arrest^110–113^. However, Hong et al. show that very high activity levels of ERKp, exceeding those that cause growth arrest, can lead to apoptosis^109^. These ultra-high ERKp levels in HEK293 and U251 cells could only be achieved by combining ERKs overexpression and the presence of tamoxifen-inducible active CR3 catalytic domain of CRAF^109^. As proposed by Hong et al., this cell death-inducing characteristic is unique to the ERKs compared to the upstream kinase effectors of the ERKp^109^.

Overall, the evidence is ample that excessive ERK activity is toxic to cancer cells with different tissue backgrounds. First, we highlight a few critical early studies among such evidence. Early evidence of ERK’s proapoptotic activity was observed in the human breast cancer cell line (MCF-7), as RAF depletion could desensitize these cells to Paclitaxel^114^. During the characterization of Phenethyl Isothiocyanate anti-proliferative effects against p53-deficient prostate cancer cell line PC-3, ERKp prolonged activation was responsible for the compound’s growth inhibitory effects^115^. Moreover, as shown in mouse embryonic fibroblasts and MCF-7, the DNA Damage (DD) caused by different stimuli, including Etoposide, could trigger ERKp activation independent of p53 but rest on Ataxia-Telangiectasia Mutant (ATM)^116^. The ERK activity was essential for DD-triggered growth arrest and apoptosis^116^. Later, it was shown that growth inhibitory and proapoptotic effects of Asiatic acid against breast cancer cell lines (MCF-7 and MDA-MB-231) were associated with the induction of ERKp^117^. In another study investigating Lauryl-gallate’s growth-suppressive and proapoptotic effect on three breast cancer cell lines MCF-7, MCF-7 ADR, and MDA-MB-231, the observed phenotype was accompanied by ERKp activity and p21-induced Cell Cycle (CC) arrest^118^. As one of the investigated cell lines was p53 mutant (MDA-MB-231) and the other possessed a multidrug-resistant phenotype (MCF-7 ADR), authors conclude that none of these conditions affects the sensitivity of cells to the tested compound^118^. Interestingly, MEK inhibition and the resulting ERKp suppression were associated with rescuing the drug effect^118^. In another study, sustained ERKp activity due to exogenous expression of active MEK1/2 led to G1 CC arrest, marked by p21^WAF^ expression^119^. Prolonged ERK activity was associated with the activation of cellular protein biosynthesis regulator p70S6K, accompanied by an increased translation of p21 protein^120^. Nonetheless, none of these studies has fully elucidated the mechanisms elicited by ERK activation to lead to the observed phenotypes. Several other studies demonstrate that reinforced ERK activity in cancer cells potentiates the cytotoxic effects of various chemotherapeutical agents (reviewed here^60,97^). In many of these studies, the MEK inhibitor compounds, if among other means, were employed to investigate the rescue effects^60,97^. Moreover, as recently highlighted by Sugiura et al.^60^ and taking into account subsequent revelations regarding MEK inhibitors off-targets and mechanisms of action, these factors could potentially undermine the precision of the abovementioned findings.

A revisit of the precedent evidence reveals an intriguing aspect of ERKp-induced cell death and senescence, as it can happen in both p53 wild-type and p53-altered backgrounds^114–120^. Numerous studies have indicated crosstalk and interactions between the p53 and ERKp, suggesting that they can influence each other’s functions and responses under specific cellular conditions. As a major tumor suppressor known as “the guardian of the genome^121^,” p53 governs safeguard mechanisms that prevent the accumulation of DNA damage and the proliferation of damaged cells^122^. The p53 protein primarily functions as a transcription factor, regulating a plethora of genes that control cell proliferation, apoptosis, DNA repair, and other cellular processes^123^. Additionally, p53 possesses multiple transcription-independent activities in cell death^124–126^, metabolism^127,128^, autophagy^129^, DNA replication^130^, and repair^131^, all of which contribute to tumor suppression and the maintenance of genomic integrity. The aberrant activation of the ERKp leads to the induction of the p53 response, which can trigger senescence^108,132–136^ or cell death^109,137^. The classical mechanism of p53 activation by oncogenes is mediated by the p14ARF protein, which sequesters the p53 inhibitory ubiquitin ligase Mdm2 (HDM2 in humans), allowing for the accumulation of the p53 protein^138^. Additionally, studies have shown that ERK can phosphorylate p53 on Ser15 under stress conditions, inhibiting its interaction with Mdm2 and promoting the stabilization of the p53 protein^139–141^. The phosphorylation of p53 by ERK2 on Thr55 was reported to induce p53 stabilization and activation in doxorubicin-treated MCF-7 cells^142^. However, it remains unclear to what extent the direct phosphorylation of p53 by ERK contributes to the induction of senescence and apoptosis in tumor cells without additional stress stimuli.

In the process of malignant transformation, cells with hyperactive ERK signaling experience strong negative selective pressure from p53^132,143–149^ which they can counteract by blunting p53 activity, often through the acquisition of TP53 mutations that entirely or partially inactivate the tumor suppressor^150^. Large-scale genomic studies have demonstrated that TP53 mutations are found in all types of cancers^151^. The frequency of TP53 genetic alterations, estimated at an average of 50%, varies significantly depending on the tumor entity, ranging from 5% in neuroblastoma to over 90% in ovarian and small-cell lung cancer^151–156^.

Somatic mutations that inactivate TP53 are predominantly observed in the later stages of tumorigenesis, indicating its role in advanced cancer progression^157^. It is estimated that 80% of genetic alterations in the TP53 gene are missense mutations^158,159^ that lead to the expression of mutant p53 proteins. These mutants can possess oncogenic properties, driving cancer progression and conferring therapy resistance^160,161^. However, the functional impact of the majority of cancer-associated TP53 missense mutations (approximately 70%) remains poorly characterized, and their role in tumorigenesis and therapy response remains unclear^162^.

TP53 mutations often co-occur with oncogenic EGFR, BRAF, and RAS mutations, but the extent of cooperation between TP53 and oncogenes may vary even within the same tumor type. For instance, in lung adenocarcinoma (LUAD), TP53 mutations are prevalent in EGFR mutant tumors but are strongly underrepresented in KRAS-driven LUAD^151,163^, indicating different paths of tumor evolution driven by different oncogenes. The intricate interplay between various oncogenes within the ERKp and its impact on the residual activity of wild-type or partial Loss-of-Function (LOF) p53, retained in tumors, which can potentially lead to tumor-suppressive effects, remains an area of ongoing investigation^162,164^. Moreover, different TP53 variants are suggested to cause distinctive LOF, dominant negative, or even GOF phenotypes, which needs to be acknowledged in further exploration of ERKp-induced lethality in different cellular contexts with distinctive TP53 status^165–168^.

### ERK pathway in light of emerging knowledge about vulnerability of cancers to replication stress targeting

In eukaryotes, transmitting genetic material to daughter cells is a crucial event and is thus tightly regulated. Cyclin-dependent kinases (CDKs) are pivotal in advancing the CC during interphase and M phases^169–171^. The CC represents a series of sequential decisions and commitments^169–171^. Each CC stage is intricately governed by a complex interplay involving CDKs coupled with E3 ubiquitin ligases like APC/C and their activating proteins^169–171^. Varied levels of CC-related proteins can establish decision windows that impact entry, prevention of re-entry, or even the deceleration of CCs^169–171^. Although tightly controlled, the CC can face undesired events due to DD, Replication Stress (RS), or spindle assembly malfunctions^169–171^. Eukaryotic cells rely on distinct checkpoints to tackle each scenario^169–171^. RS emerges when diverse factors slowdown or stall the replication fork. DD and RS differ, but the latter can trigger DD as the collapse of the stalled fork can lead to double-strand breaks^169–172^. RS checkpoints function to avoid RS-induced DNA damage response, while DD checkpoints are meant to prevent the accumulation of DNA damage and thereby protect cells from subsequent complications^170^. A cancer’s hallmark is uncontrolled proliferation, hampering apoptosis and avoiding long-term CC exits^34^. Oncogenes like RAS and MYC can negatively affect DNA replication licensing and firing, inciting RS^169,170,172^. Deregulation of DD and growth pathways due to cancer-related mutations causes excessive S phase entry and subsequent RS. While a temporary check-out from the Mitosis/entry is possible, leaving the CC is not favored^169–172^. Cancer cells hold a higher basal level of RS vs. normal cells^170^. The severity of the damage and the context determines the cell fate, which can be apoptosis, quiescence, or senescence. It deserves to be mentioned that DD checkpoint responses can lead to a temporary exit or, as opposed to RS, an eternal exit of the CC^169–172^. However, DD responses and RS are so intertwined that, ultimately, RS can lead to such events^169–172^. Curiously, cancer cells highlight a distinct approach to DD and RS checkpoints. DD checkpoints are often surrogated in cancer; cancer cells tolerate defects in DD response and perhaps even benefit from such a compromise in favor of increased selection pressure, all while sidestepping unfavorable exits from the cell cycle^169–172^. Conversely, RS checkpoints are somehow intact^169–172^. Indeed, cancer cells are more sensitive to DD responses than RS responses^170^. Cancer cells tolerate and even favor some Chromosomal instability (CIN) levels^170^. In contrast, they cannot tolerate excessive CIN, which can be caused by excessive RS and DD, leading to catastrophic mitotic defects, loss-of-essential genes, and cell death^170^. Therefore, cancer relies on RS checkpoints to avoid too much CIN. Consequently, targeting RS tolerance in cancer holds promise as a viable cancer therapy approach^169–173^. Of note, studies have demonstrated that elevated oncogenic RAS activity can trigger RS by ubiquitously enhancing cellular transcription events^174–176^. This augmented transcription activity increases the likelihood of collisions between replication and transcription processes^174,176^. In addition to RS induction, oncogenic RAS (HRAS^G12V^) can circumvent the p53 activity, thereby sensitizing cancer cells to RS-inducing compounds^176^.

### Withdrawing ERK pathway inhibitors from addicted cells: lethal consequences of excessive pathway activity

There are several pieces of evidence that ERK-related cancer cells can tolerate upregulation of the ERKp only in the presence of ERKp inhibitors. Resistance mechanisms against ERKp inhibitors predominantly involve ERKp effectors and regulators^30,31,81,177–181^. Different RAF and MEK inhibitors can trigger clonal expansion of drug-tolerant cells, which maintain a proliferative advantage, perhaps preferably in the presence of inhibitors by virtue of enhanced ERKp activity^182,183^. This elevated activity could be lethal upon inhibitor cessation, potentially resulting in ERK-related cellular toxicities^183^. It is suggested ERKp inhibitors can create a window upon drug removal, in which cells lose their fitness advantage gained during drug treatment and may even experience growth disadvantage due to excessive ERKp activity and adaptive switching^182,184^. In an elegant study by Kong et al. conducted in Peeper lab^185^, a CRISPR knock-out screen of melanoma cells resistant and addicted to BRAFi revealed a phenotypic switch dependent on ERK2 kinase and JUNB and FRA1 transcription factors accompanied by suppression of microphthalmia-associated transcription factor (MITF)^185^. The ERK2 dependency of the observed phenotype was supported by in vivo and clinical findings^185^. This dependency was also observed in lung cancer cells resistant and addicted to EGFRi^185^. The addicted Melanoma cells experienced grave cell death upon withdrawal from BRAFi, which could be rescued by ERK2 targeting or restoring MITF activity^185^. Another intriguing aspect of this study was that the authors opted to investigate the intermittent drug treatment with the chemotherapeutic agent dacarbazine, and they showed BRAFi-addicted melanoma cells were sensitized to this compound accompanied with MITF inhibition^185^.

Aissa et al. elegantly showed at the single-cell level that drug-resistant EGFR mutant lung cancer cell clusters exhibited markers indicative of activated ERKp^180^. Recent work by Nuria Gutierrez-Prat et al.^186^ reported similar findings concerning ERK and MITF dependency on drug withdrawal toxicity. Moreover, the authors find that the knock-down of DUSP4, an ERK phosphatase and negative regulator, was lethal by causing excessive ERK activity^186^. This effect was observed not only in melanoma cells addicted to inhibitors of the ERKp but also, intriguingly, in drug-naïve cells^186^. Xue et al. in Piro Lito’s lab found the link between oncogenic BRAF protein dosage in cells, the depth of ERKp inhibition and the related resistance mechanisms. In their patient-derived xenograft lung cancer and melanoma models, they discovered the more robust the ERK inhibition is, the higher the oncogene dosage required for cells to retain proliferation advantage in the presence of inhibitors^183^. They proposed a fitness threshold model, suggesting that cells treated with regimens with a higher threshold, such as upon combination of RAFi, MEKi, and ERKi vs. ERKi monotherapy, might face a disadvantageous outcome due to sustained and excessive ERKp activation when the drug treatment is stopped^183^.

Some preclinical and early clinical evidence suggested the benefits of intermittent treatment with RAFi and MEKi vs. sequential treatments, in particular in melanoma^182–184,187–197^. This evidence laid the foundation for clinical trials examining intermittent treatment regimens in individuals with BRAF mutant melanoma^198,199^. Contrary to the expectations, these trials did not show any overall survival benefits from the intermittent therapies, and even worse progression-free survival outcomes were reported upon intermittent treatments^198,199^. Nevertheless, it is still uncertain whether those intermittent therapies meant to produce that high fitness threshold as recommended by Xue et al. to cause selection disadvantages in tumor cells during drug removal effectively.

### Mutual exclusivity of highly activating variants of BRAF, KRAS and EGFR oncogenes in cancer: induction of synthetic lethality or senescence

In 2006, Carlotta Petti et al.^200^ showed that synthetic expression of NRAS^Q61R^ oncogene in a metastatic melanoma clone, which natively harbored the mutually exclusive BRAF^V600E^ oncogene, resulted in senescence. Later, Cisowski et al. discovered that co-expression of BRAF^V600E^ and KRAS^G12D^ under their endogenous promotors provides a selective disadvantage compared to single oncogene expression in mouse lung cells^53^. The decrease in tumor burden in double oncogene-expressing tumors was associated with hyperactivated ERK and AKT signaling and a decrease in proliferating cells^53^. Further analysis demonstrated enhanced β-galactosidase expression and increased p15, p16, and p19 levels upon oncogenes co-expression, suggesting that double oncogene-expressing cells become senescent^53^. Meanwhile, Unni et al. exogenously induced the expression of KRAS^G12V^ or EGFR^L858R^ in EGFR^EX19Del^ and KRAS^G12C^ LUAD cell lines, respectively^43^. They observed decreased cell viability, indicating that mutant KRAS and EGFR co-expression are not tolerated in cells^43^. Additionally, they generated genetically engineered mice with co-induction of KRAS and EGFR mutants in lung epithelium. The established lung tumors did not grow faster than those harboring only one of the oncogenic mutations did^43^. Further analysis indicated that only one of these oncogenes could be activated in the tumor cells^43^. Furthermore, Unni et al. revealed that DUSP6 prevents ERK activity from exceeding critical thresholds in EGFR and KRAS mutant cell lines^201^. They found that targeting DUSP6 reduced cell viability due to unleashing the excessive and toxic levels of RAS-mediated ERK activity in cancer cells harboring mutations in EGFR and KRAS^201^. Markedly, Ambrogio et al. showed that conditional induction of an EGFR^L858R^ allele in KRAS^G12V^ knock-in mouse LUAD models led to decreased tumor burden, increased mice survival, and reversible cell toxicity in remaining tumor cells^202^. The latter could further be recovered through ERKp activity reduction^202^. Of note, all these consequences were associated with hyperactivation of ERKp signaling^43,201,202^.

Harold Varmus, a Nobel Prize laureate, and his colleagues, who played key roles in some of the aforementioned studies, articulated and championed the intriguing concept that, unlike how previously considered, not the redundancy of functions but synthetic lethality or senescence could lie behind mutual exclusivity of certain oncogenes in cancer^56^.

We argue that the pathway redundancy and synthetic lethality, senescence, or any other constraining phenomenon should not necessarily be seen as conflicting scenarios. One can consider that pathway redundancy and synthetic lethality could both play roles in explaining mutual exclusivity. When two proteins with overlapping functions in a pathway are excessively active, the likelihood of both events being mutually exclusive within a cancer cell is increased.

On co-induction of activating EGFR and BRAF events in the same cell, it has been shown that exogenous expression of wild-type EGFR in a Melanoma cell line with native BRAF^V600E^ leads to decreased proliferation of these cells in a dose-dependent manner, in-vitro and in-vivo^203^. The slowdown in proliferation was associated with cellular senescence as suggested by hypophosphorylation of RB1 and induction of CDKN1A, CDKN1B, and Beta-galactosidase^203^. Concerning mutant EGFR and mutant BRAFs, two studies have been inspired so far by the emergence of BRAF^V600E^ in treatment-refractory EGFR^L858R^ lung cancers as they become resistant to EGFR-targeted therapies^177,179^. In one study, exogenous expression of BRAF^V600E^ in a polyclonal pool of EGFR mutant lung cancer cells led to no differences in cell proliferation and cell death rate compared to empty vector control^177^. Of note, the BRAF^V600E^ protein levels and mRNA levels showed an indispensable increase only in the presence of an ERKp inhibitor, suggesting that in the polyclonal pool, cells with BRAF^V600E^ induction are not clonally expanded unless the ERKp activity is suppressed^177^. As such, further investigation is required to determine the existence and the mechanism behind such clonal disfavor. Overall, the findings of these two studies align with the results of two independent GOF CRISPRa screens in Vemurafenib-treated A-375 cells (BRAF^V600E^), showing that overexpression of EGFR, among others, is a resistance mechanism to BRAFi^204,205^.

Three BRAF mutational classes^26^ include Class I variants, like BRAF^V600E^, which often function independently of upstream effectors as constitutively active monomers. Class II variants, exemplified by BRAF^G469A^, activate the ERK pathway independently of RAS and CRAF as active homodimers. Class III involves kinase-dead BRAF mutants activating the ERK pathway through RAS-dependent allosteric transactivation of CRAF.

Cancer-relevant KRAS mutations are classified into three categories^73^ based on their impact on KRAS protein functions: Class I (Hydrolysis) includes mutations leading to the loss-of the GTP-hydrolyzing feature of KRAS, Class II (Exchange) involves mutations causing a gain in KRAS Exchange function facilitated by Guanine Nucleotide Exchange Factors, and Class III (Hybrid) encompasses mutations affecting both functions.

A recent classification of EGFR mutations^24^ considers both the structural effects of mutations on the EGFR protein, specifically its drug-binding pocket (DBP), and the implications of mutations on drug response. Accordingly, one of the EGFR mutational classes is Classical-like (relevant to this writing), where mutations like L858R have minimal impact on DBP and the affinity for corresponding Tyrosine Kinase Inhibitors.

Recently, the variant-specific landscape of mutual exclusivity among BRAF, KRAS, and EGFR mutations in cancer has been unraveled^206^. We learn which oncogenic variants can co-occur in the same cancer sample while certain driver events are mutually exclusive. The authors conclude that class I BRAF(in line with another recent report^206,207^), Hydrolysis KRAS^206^, and classical-like EGFR^206^ class mutations are less likely to co-occur. When they dissected the analyses into variants, they discovered novel instances of mutual exclusivity involving unconventional yet common oncogenic variants. They showed that specific classical-like EGFR and BRAF mutations, often the most frequent ones, are mutually exclusive in human cancer^206^.

### Leveraging oncogenes mutual exclusivity for precision oncology: a target discovery framework for RTK/RAS/RAF pathway agonism

**Fig. 4:**
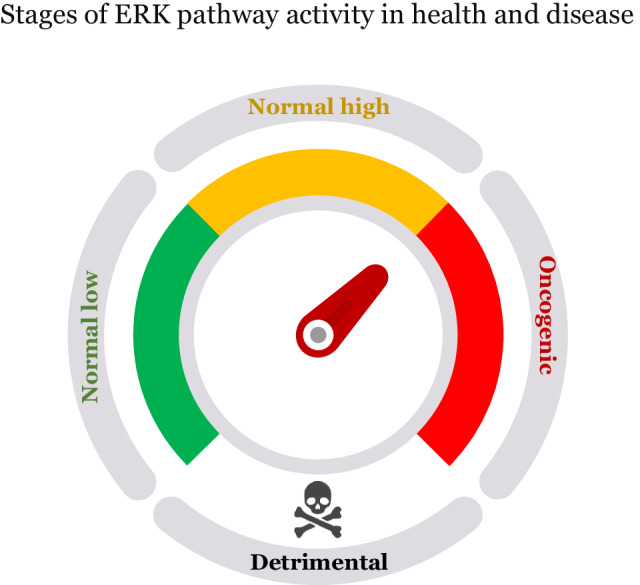
Simplified schematic illustrating the stages of ERKp activity in health and disease, and upon explored and suggested therapeutic targeting. This schematic is oversimplified and does not acknowledge the spatiotemporal context of ERK activity and regulation. The figure was generated using a template provided in https://www.slideegg.com.

Reinforced activation of the ERKp could serve as a therapeutic strategy for ERK-associated cancers. As previously explained, these cancers exhibit susceptibility to elevated ERKp activity beyond conventional oncogenic levels. Importantly, this vulnerability can be selectively targeted because normal cells and ERK-associated cancer cells differ in their baseline ERKp activities. Thus, reinforcing ERK activation to levels intolerable for cancer cells may not necessarily push normal cells beyond the tolerable threshold (see Fig. 4).

At first glance, the primary concern with this approach is the potential undesired activation of the ERKp in non-cancerous tissues. Interestingly, this potential adverse event is not unfamiliar in precision oncology. Patients with BRAF^V600E^ mutant cancers have been treated with various RAF inhibitors for years, initially as single agents and later combined with MEK inhibitors. Type I RAF inhibitors like Vemurafenib and Dabrafenib can cause paradoxical ERKp activation in non-cancerous cells with wild-type B/CRAF. Some patients develop benign teratomas like keratoacanthoma, but most do not^26,208,209^. Adding MEK inhibitors to therapy can significantly reduce the occurrence of such adverse events, although MEK inhibition is also associated with toxic effects^210^. Furthermore, predictive markers and signatures can help identify patients at risk of these adverse events. On the other hand, developing selective ERKp activators with exclusive effects on cancer cells could mitigate this challenge from the outset.

Our proposed model recognizes three non-detrimental and two detrimental stages and four thresholds of ERKp activity (Fig. 4). The “normal low” occurs when cells are at rest. The “normal high” occurs when cells are in proliferating status or are stressed due to intrinsic or extrinsic signals. ERKp activity spatiotemporally surpassing “normal high” physiological levels enters the “oncogenic window.” Levels below the “normal low” and above the “oncogenic window” can be detrimental. For any potential therapy, it will be necessary to set pathway activity exceeding the “oncogenic window” and entering the detrimental stage in the target cancer cells. Ideally, the therapy should not push pathway activity beyond “normal high” levels in normal cells, adhering to the Goldilocks principle.

**Fig. 5:**
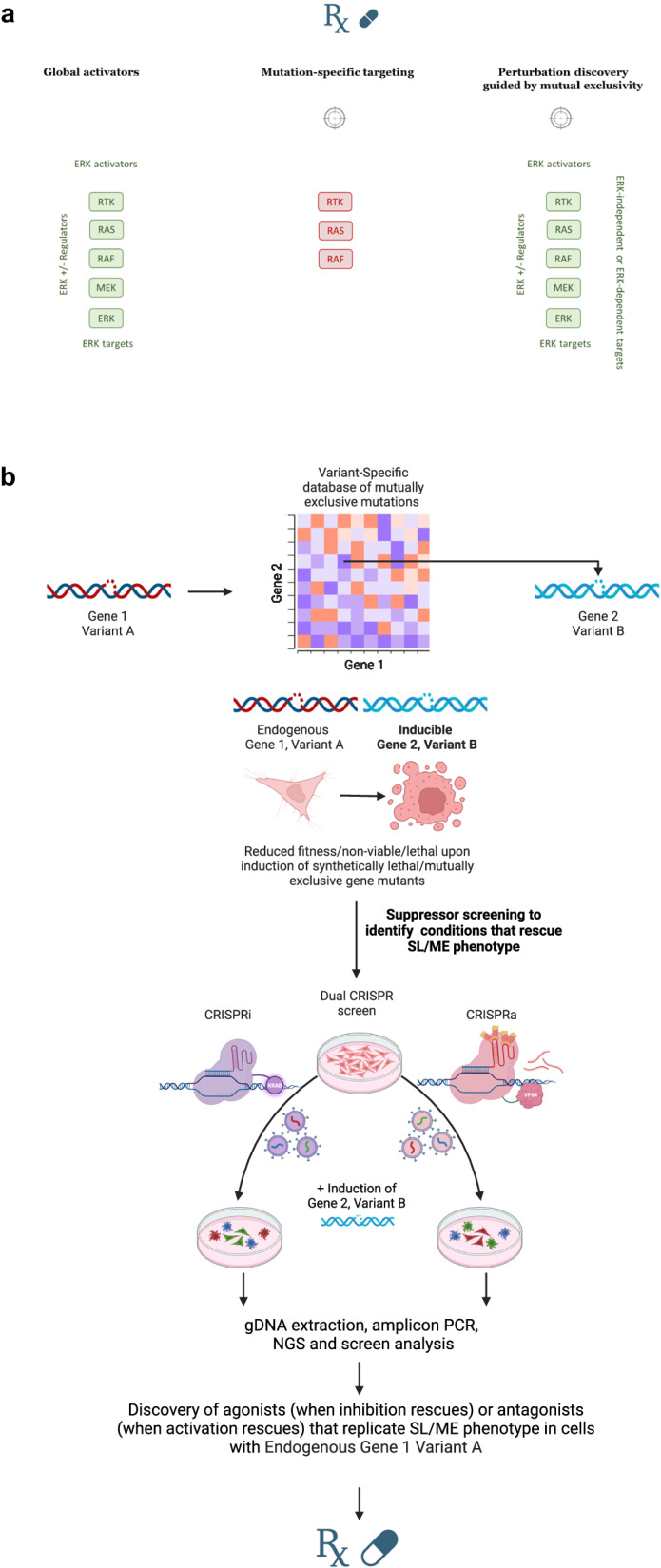
Three Perturbations (P1-3) to reinforce the already activated ERK pathway activity in related cancers as a therapeutic approach. **a** Three suggested approaches to target the vulnerabilities to reinforced RTK/RAS/RAF pathway activation in affected cancers. **b** Framework for P3 target discovery is displayed in a more detailed manner. Figure generated in Biorender.

This discussion will not delve into the chemistry and structural aspects of potential therapeutics, including small molecule activators, monoclonal antibodies, monobodies, etc. We propose three strategies to reinforce the ERKp or ERK-independent signals for detrimental effects (Fig. 5a, b).

#### Perturbation 1 (P1)-global activators

Potential therapeutics activate the ERKp through various means, such as ligand-mimetic molecules targeting EGFR, irrespective of the specific oncogenic mechanism of the treated cell. While not selective towards their direct targets, these therapeutics can exert selective detrimental effects on cancer cells. Recently, Xu et al. from Deng’s lab have introduced and characterized the first-in-class small molecule KRAS agonist that activates both the wild-type and mutant KRAS molecules and has shown selective effects towards KRAS mutant lung cancer in-vitro and in-vivo^57^. Another tactic involves directly targeting the ERK1/2 proteins. Exciting research in Sugiura’s lab and colleagues has explored ERKp agonists and lead compounds that show selective activity against ERK-associated cancer cell lines compared to normal cells^58–60^. Unsupervised screens involving perturbations to activate different activating effectors of the ERKp can be applied to find the most effective targets.

#### P2-Mutation-specific targeting

Targeting protooncogenes like BRAF^V600^, EGFR^L858R^, or KRAS^G12V^ with mutation-specific agonists can elevate basal ERK activity beyond the oncogenic window in cancer cells, leading to events like apoptosis or senescence. These agonists should ideally be selective (if they ever could be) against oncogenic variants of the targeted- versus the wild-type proteins, aligning with Paul Ehrlich’s *magic bullet* concept. It deserves to be mentioned that as oncogenes, like any other protein, have a threshold of conformational stability; it cannot be ruled out that oncogene agonists might end up destabilizing and further orchestrating protooncogene degradation processes before even exerting the envisioned effects.

#### P3-Unsupervised target discovery and targeting aided by the variant-specific landscape of mutual exclusivity among BRAF, EGFR, and KRAS oncogenes in human cancer

We discussed how induction of mutually exclusive BRAF, EGFR, and KRAS oncogenes could be detrimental in affected cells. Nature itself is presenting these scenarios to us. While all the hypothesis-driven approaches mentioned above show promise for further investigation, we would like to underscore an unbiased target discovery approach guided by the uncovered variant-specific landscape of mutual exclusivity among BRAF, EGFR, and KRAS oncogenes. *Oncogene overdose*^55^, mimicking the co-expression of EGFR and KRAS oncogenes^56^, *paradoxical intervention*^61^, and *ERK-Dependent Apoptosis*^60^ as therapeutic models proposed by different research groups share fundamental similarities. Nevertheless, they vary in certain specific details. The recent unveiling of the variant-specific landscape of mutual exclusivity among ERKp oncogenes introduces a new layer of complexity to existing concepts. This newfound complexity offers insights into the hyperactivation of specific signaling pathways that certain cancer cells circumvent to avoid most hostile conditions.

**Fig. 6:**
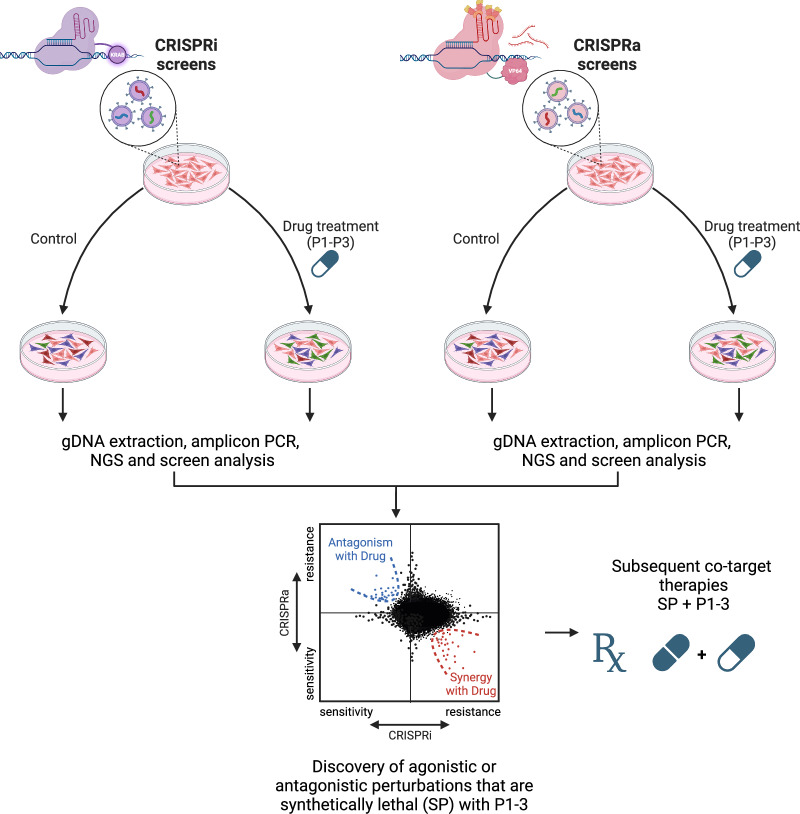
Suggested approach for co-target discovery to more profoundly target the vulnerabilities to reinforced hyperactivation in ERK-associated human cancer. Figure generated in Biorender.

Consequently, replicating these mutually exclusive scenarios could lead to highly effective yet personalized therapies tailored for each oncogenic variant (BRAF, KRAS, and EGFR). Therefore, in the proposed model, the toxic effects of two oncogenes’ joint expression would be limited to specific variants, or at least more likely in those mutually exclusive scenarios. Consequently, in our proposed model, both target (Fig. 5b) and co-target discovery (Fig. 6) can be a matter of precision. Considering this, our model suggests that the initial point of target discovery and potentially resulting therapy, for instance, for the BRAF^G469V^ variant, may differ from that of BRAF^V600E^. Unbiased approaches, such as bulk RNA sequencing and single-cell RNA sequencing, can help explore differentially expressed genes and altered pathways resulting from the induction of mutually exclusive BRAF, EGFR, and KRAS oncogenes. Genetic screens such as CRISPR screens, especially dual LOF and GOF screens (i.e., CRISPRi and CRISPRa), may identify the signaling nodes that govern the response and dictate cell fate when mutually exclusive oncogenes are co-induced. These screens are intended to elucidate targeting approaches that phenotypically replicate the co-induction of two synthetically lethal and mutually exclusive gene mutations. Merely Droup-out screens like KO screens may fall short in identifying negative regulators of the ERKp as potential signaling nodes.

Of note, this approach can ideally be tailored to each tumor. For example, for target discovery of cancer cells with BRAF^V600E^, one can refer to the variant-specific landscape of mutual exclusivity database^207^ and find the most mutually exclusive scenario with BRAF^V600E^, and as such, design a model with inducible expression of KRAS^G12D^ or EGFR^L858R^ in BRAF^V600E^ background. In a suppressor CRISPR screen, those genes whose activation or inhibition will lead to the rescue of senescence or synthetic lethality would be the governing nodes and potential targets whose antagonism (when activation rescues) or agonism (when inhibition rescues) replicate the synthetically lethal co-induction of these mutually exclusive oncogenes (Fig. 5b). This approach widens the targeting possibilities and may fit both the Goldilocks principle and the *magic bullet* concept. Besides, this approach may fit the concept of precision oncology.

It is crucial to emphasize that the RTK/RAS/RAF effectors, the inhibitors designed to target them, and their regulators can demonstrate functions and interactions independently of one another, their kinase activity, or even the ERKp itself^211–213^. Further investigation exploring the RTK/RAS/RAF agonism as a therapeutic approach must address whether these functions can be leveraged to enhance oncogenic activity beyond tolerable levels for potential therapeutic benefits or, conversely, whether they might counteract such a strategy. Therefore, any proposed approach to exploit the susceptibility of ERK-associated cancers to hyperactivation of these effectors should consider this notion. If the desired detrimental effect would be ERK-independent, the P1 might fail to address it.

Widespread and highly active oncogenic variants (gene 1), in mutually exclusive relationships with other variants (gene 2), align with the third approach for target discovery (Fig. 5a, b: P3). Conversely, cells harboring oncogenes with co-occurring tendencies^206^ (variants of gene 1 and gene 2) may align with P1 and P2 (Fig. 5a).

## Combined targeting

Cancer targeting resembles a time loop: as monotherapies are developed, resistance emerges, driving the search for more effective combinatorial treatments that not only offer a stronger initial impact but may also delay the development of further resistance. Therefore, it would be wishful thinking to predict that P1-3 would be exempt from the rise of resistance. As such, concurrent, sequential, or intermittent combinatorial treatments can be explored depending on the ultimate phenotypes exerted by putative therapeutics. Based on the evidence revisited in this writing, opportunities for combinatorial treatments with chemotherapeutics can be studied, whether as double-punch or one-two-punch with senolytics. Suppose ERK agonism would lead to immune evading phenotypes. In that case, a *one-two-punch* model can be explored to address whether, after ERK agonism, the remaining tumor could be sensitized to immune checkpoint inhibitors. The impact of ERK agonism on cancer stem cells and in-parallel or subsequent sensitivity of this subpopulation to chemotherapy or stem cell-targeted therapies is also an avenue worthy of exploration. Notably, P1-P3 hold promise to be explored in combination with therapeutics targeting the RS tolerance.

For co-target discovery, we propose that the initial conditions and ongoing perturbations will be pre-determined using one of the P1-P3, as we discussed for single-target discovery (Figs. 5a, b and 6). In this context, dual loss- and GOF screens (e.g., CRISPR screens) will prove valuable in identifying co-targets when ERK-associated cancer cells undergo the pre-determined ERK-activating perturbation (P1-P3). This approach aims to uncover **s**ynthetically lethal co-**p**erturbations (SP) with P1-3, enhancing the detrimental effects to the levels warranting higher fitness threshold^191^ and the emergence of tumor-suppressive drug resistance mechanisms^62^. In this respect, our proposed model differs from the paradoxical intervention model, where co-perturbations are identified within the context of hypothesis-driven constant perturbation, such as stress-inducing agents^61,62^. Consequently, we envision unbiased target discovery for both the discovery of the ERK-activating signaling nodes that govern the detrimental effects of excessive ERKp activation and the subsequent co-target discovery phase (P3 combined with SP, see Fig. 6). Therefore, our model may precisely recapitulate the most hostile conditions cancer cells are avoiding, which are the co-induction of specific oncogenic scenarios.

## Conclusions

Despite ample evidence that cancer cells are sensitive to excessive RTK/RAS/RAF pathway activity, this approach has not been widely explored. Perhaps years of relentless efforts to develop efficient ERK inhibitory treatments have established a psychological barrier within us, the scientific community, which, if not overcome, could become a dogma.

Learning from the past, we humbly recommend that all the proposed strategies in this writing undergo unbiased preclinical exploration without any premature preference for a specific strategy over the others.

Despite the primary focus of this writing being exclusively on the ERKp, the mutual exclusivity of cancer-related genetic events offers a wealth of information regarding unexplored synthetic lethality scenarios. Harnessing these scenarios for therapeutic purposes could open new horizons in targeting cancer-related vulnerabilities. Apart from targeting oncogenes, this approach can also encompass events related to tumor suppressors, such as p53, especially when cancer cells remain sensitive to restoring the related functions. Exploring these approaches and identifying targets demands a collaborative effort involving collective benchwork and shared intellectual contributions.
